# Reducing Tacrolimus Levels to Improve Cognitive Function in Kidney Transplant Recipients

**DOI:** 10.34067/KID.0000000000000454

**Published:** 2024-07-25

**Authors:** Miguel Bigotte Vieira

**Affiliations:** Nephrology Department, Hospital Curry Cabral, Centro Hospitalar Universitário de Lisboa Central, Lisbon, Portugal and NOVA Medical School, Universidade NOVA de Lisboa, Lisbon, Portugal

**Keywords:** kidney transplantation, organ transplant, transplantation

Cognitive impairment is the loss of brain functions including concentration, attention, executive function, verbal fluency, learning, and memory beyond what is expected for age. It ranges from mild cognitive impairment to dementia.^1^ The prevalence of cognitive impairment in patients with CKD ranges from 10% to 40% and is higher in older patients with ESKD.^2^ The duration of kidney disease, the decrease in GFR, and the presence of albuminuria are associated with the development of cognitive impairment.^1,2^ In patients with CKD, cognitive impairment is associated with decreased quality of life, poorer adherence to treatment plans, longer hospital stays, lower likelihood of being listed for kidney transplantation, increased time until transplant listing, and increased risk of death.^2–4^ The main contributor to poorer cognitive function in these patients is probably cerebrovascular disease. However, other factors such as impaired clearance of uremic metabolites, depression, sleep disturbance, anemia, and polypharmacy may also contribute.^2^ Several strategies have been proposed to improve or delay cognitive impairment, including modifying cardiovascular risk factors that contribute to vascular disease, avoiding sedating medication, evaluating sleep hygiene, enhancing social support networks, encouraging regular exercise, and mental stimulation.^2^ To enhance treatment adherence, it is recommended that medication management plans are established, check-ins are conducted frequently, clear written instructions are provided, and active involvement of health care professionals is ensured. Reducing BP and albuminuria through the use of angiotensin-converting enzyme inhibitors or angiotensin receptor blockers may potentially attenuate cognitive decline, albeit to a modest extent.^2^ Although data are lacking in patients with CKD, preliminary evidence suggests that sodium–glucose cotransporter-2 inhibitors may improve cognitive impairment in patients with type 2 diabetes mellitus.^5^

Evidence is insufficient to support any modification in dialysis prescription or dialysis delivery to prevent cognitive impairment. However, in patients experiencing recurrent episodes of intradialytic hypotension, it might be reasonable to reduce the dialysate temperature.^2^ Although cognitive performance typically worsens as kidney function declines, kidney transplantation seems to result in enhanced cognitive function in children and adult recipients, preemptive and postdialysis recipients, and living and deceased donor kidney recipients.^2^ This suggests that dialysis modalities do not yield cognitive advantages comparable with having a functional kidney. Short-term improvements in cognitive function post-kidney transplantation are observed in both frail and nonfrail recipients, although frailty is linked to medium-term cognitive decline.^6^ There are different potential explanations for cognitive function improvement after transplantation. Successful transplantation restores crucial functions of the kidney, such as filtration, secretion, tubular function, hormonal balance, and clearance of medications. In addition, transplantation eliminates the need for dialysis and the associated complications that may promote cognitive impairment, such as hemodynamic shifts, use of anticoagulation that may predispose to microbleeds, and intermittent solute clearance, particularly with hemodialysis.^2^ Furthermore, depression tends to improve after kidney transplantation, which is likely to enhance performance in neuropsychological tests.^7^

Cognitive impairment in kidney transplant recipients is viewed as a clinically relevant problem that is often underestimated and goes unrecognized.^3^ Unfortunately, despite the improvement in cognitive function after kidney transplantation, average cognitive performance remains below that of the non-CKD population. This may indicate irreversible brain alterations. It is also possible that immunosuppressive medications hinder cognitive function improvement after kidney transplantation. The administration of high doses of corticosteroids, employed for induction and treatment of rejection, is associated with cognitive impairment, along with psychotic symptoms.^7^ Calcineurin inhibitors (*e.g*., cyclosporin, tacrolimus) cause endothelial dysfunction and are inherent vasoconstrictors. ESKD patients present an increase in cerebral blood flow, which occurs probably because of inflammation, vascular disease, and disruption of cerebral autoregulation. Nevertheless, cerebral blood flow decreases after kidney transplantation to values lower than those observed in healthy people. Decreased cerebral blood flow is associated with cognitive impairment.^8^ Although uncertain, it is possible that calcineurin inhibitors–induced vasoconstriction contributes to this phenomenon.^7^

In the current issue of *Kidney360*, Tariq *et al.*^9^ reported an open-label, single-center, prospective pilot study of 39 kidney transplant recipients with stable allograft function on high-dose immediate release tacrolimus and an antimetabolite, with or without prednisone. Patients in the intervention group were started on everolimus, tacrolimus was reduced from a trough level of 7–10 to 3–5 ng/ml, and the antimetabolite was stopped. Study assessments included a noncontrast magnetic resonance imaging to measure cerebral blood flow and a battery of standard neuropsychological tests to evaluate cognitive function at baseline and at 12 weeks. Briefly, there was an increase in cerebral blood flow across different brain regions and an improvement in cognitive function.

This is an interesting study that offers insight into a possible intervention to improve cognitive function in kidney transplant recipients. To date, no treatment has been approved by regulatory agencies as effectively slowing or preventing cognitive impairment in kidney transplant recipients.^3^ Note that in this study, it is not clear whether the observed changes were due to vasoconstriction induced by tacrolimus, another direct effect of tacrolimus, the discontinuation of the antimetabolite, a beneficial effect of everolimus, or other unidentified factors. Everolimus is a mammalian target of rapamycin inhibitor used as an immunosuppressant in transplant recipients. It has previously been shown to positively affect cognitive functions in animal models of dementia, and a potential beneficial effect on cognition cannot be ruled out.^1^

As criteria for kidney donation and transplant listing are broadened, the kidney transplant population increases and gets older. Identifying mild cognitive impairment early is crucial as it can serve as a precursor of dementia. Interventions aimed at slowing cognitive decline may be more effective at this stage compared with after the onset of overt dementia.^7^ Despite its limitations, including the lack of randomization, small sample size and short-term follow-up, the notable investigation by Tariq *et al.* is a proof-of-concept longitudinal study with detailed cognitive assessments.

Future research should test different tacrolimus trough levels, ideally including a calcineurin inhibitor–sparing regimen, in a larger study with longer follow-up. It would also be important to assess the isolated effect of antimetabolite discontinuation and everolimus introduction. Immunosuppression optimization may be a promising strategy to improve cognitive function after kidney transplantation but needs to be carefully balanced against the risk of rejection and renal graft function decline.
